# The N-Terminus of Vps74p Is Essential for the Retention of Glycosyltransferases in the Golgi but Not for the Modulation of Apical Polarized Growth in *Saccharomyces cerevisiae*

**DOI:** 10.1371/journal.pone.0074715

**Published:** 2013-09-03

**Authors:** Jia-Wei Hsu, Lin-Chun Chang, Li-Ting Jang, Chun-Fang Huang, Fang-Jen S. Lee

**Affiliations:** 1 Institute of Molecular Medicine, College of Medicine, National Taiwan University, Taipei, Taiwan; 2 Department of Medical Research, National Taiwan University Hospital, Taipei, Taiwan; Université de Nice-CNRS, France

## Abstract

Vps74p is a member of the PtdIns(4)P-binding protein family. Vps74p interacts with Golgi-resident glycosyltransferases and the coat protein COPI complex to modulate Golgi retention of glycosyltransferases and with the PtdIns(4)P phosphatase Sac1p to modulate PtdIns(4)P homeostasis at the Golgi. Genetic analysis has shown that Vps74p is required for the formation of abnormal elongated buds in *cdc34-2* cells. The C-terminal region of Vps74p is required for Vps74p multimerization, Golgi localization, and glycosyltransferase interactions; however, the functional significance of the N-terminal region and three putative phosphorylation sites of Vps74p have not been well characterized. In this study, we demonstrate that Vps74p executes multiple cellular functions using different domains. We found that the N-terminal 66 amino acids of Vps74p are dispensable for its Golgi localization and modulation of cell wall integrity but are required for glycosyltransferase retention and glycoprotein processing. Deletion of the N-terminal 90 amino acids, but not the 66 amino acids, of Vps74p impaired its ability to restore the elongated bud phenotype in *cdc34-2/vps74*Δ cells. Deletion of Sac1p and Arf1p also specifically reduced the abnormal elongated bud phenotype in *cdc34-2* cells. Furthermore, we found that three N-terminal phosphorylation sites contribute to rapamycin hypersensitivity, although these phosphorylation residues are not involved in Vps74p localization, ability to modulate glycosyltransferase retention, or elongated bud formation in *cdc34-2* cells. Thus, we propose that Vps74p may use different domains to interact with specific effectors thereby differentially modulating a variety of cellular functions.

## Introduction

Genetic screening methods in yeast are powerful tools that facilitate gene discovery and functional characterization. The *VPS74* gene has been isolated from several different genetic screens. *VPS74* was isolated in a mannan-defective mutant screen [1,2], as a *YPT6*-interacting gene in the large-scale global mapping of the yeast genetic interaction network [3]. Furthermore, *VPS74* contributes to apical growth, as determined in a directed allele replacement technology (DART) screen [4].

Mannan-defective mutants (*mnn* mutants) of *Saccharomyces cerevisiae* were originally isolated based on their modified cell wall mannan structures. The last *MNN* gene identified among *mnn* mutants was *MNN3*, which is a synonym of *VPS74* [5]. Both N-linked and O-linked mannosylation events are affected, and the carbohydrate chains of mannosylated proteins are shortened in the *mnn3*Δ mutant. These phenotypes could not be explained by the loss of a single mannosyltransferase enzyme; thus, the *MNN3* gene product might play a regulatory role that simultaneously modulates the activities of multiple mannosyltransferases [2,5]. *VPS74* was also isolated in a screen designed to identify yeast genetic networks that are synthetically lethal with *YPT6* and in a screen for dosage suppressors of the lethality resulting from the deletion of *SFT1*. Ypt6p is a Rab family member that regulates intra-Golgi and endosome-to-Golgi trafficking [3]. Sft1p is an essential Golgi-resident SNARE protein that is required for retrograde trafficking within the Golgi. Genetic interaction studies of Vps74p with these genes have suggested that Vps74p may participate in the retrograde transport involving the Golgi complex [6].

Recent studies have reported that Vps74p is required for the proper localization of several Golgi glycosyltransferases [6]. These studies found that GFP-tagged Vps74p is both localized to the Golgi complex and distributed diffusely in the cytoplasm. Deletion of *VPS74* results in the mislocalization of Golgi-resident glycosyltransferases, including Kre2p, Mnn2p, Mnn5p, Mnn9p, Och1p, and Ktr6p [6]. X-ray crystallographic analyses of the Vps74p structure have revealed that Vps74p forms a tetramer in solution. Further study has shown that this tetramerization contributes to the association of Vps74p with the Golgi and is crucial for the binding of Vps74p to a pentameric sequence motif at the cytoplasmic tails of glycosyltransferases [7]. Vps74p binds directly to coatomer (coat protein; COPI), the vesicle coat complex that mediates retrograde trafficking [6]. These studies proposed that Vps74p binds to and modulates the packaging of Golgi-resident glycosyltransferases into COPI-coated vesicles, mediating their recycling back to the Golgi. These findings both described the phenotypes resulting from *mnn3*Δ mutations and suggested a role for Vps74p in retrograde Golgi transport.

Interestingly, *VPS74* was also isolated in a large-scale screen to identify genes that alter the elongated bud morphology induced by a prolonged apical growth phase in *cdc34-2* cells at a restrictive temperature [4]. The replication of *S. cerevisiae* by budding is a two-phased process that consists of an apical growth phase and an isotropic growth phase. Apical growth occurs immediately after bud emergence for a brief period in the G1 phase. During this period, secretion and cell wall deposition are restricted at the distal tip of the growing bud. The isotropic growth phase is initiated upon entry into the M phase. During isotropic growth, the deposition of materials and growth are no longer focused at the bud tip but rather occur throughout the entire bud surface [8]. The cyclin-dependent kinase Cdc28p modulates the transition from apical to isotropic growth by promoting apical growth upon activation by G1 cyclins. When activated by mitotic cyclins (Cln), Cdc28p promotes isotropic growth [9,10]. Cdc34p is an E2 ubiquitin-conjugating enzyme that facilitates the degradation of the G1 cyclins Cln1p and Cln2p and the G2 cyclin/cdk inhibitor Sic1p [11,12,13]. Yeast cells harboring the temperature-sensitive allele of *CDC34* (*cdc34-2*) cannot enter isotropic growth and formed multiple elongated buds when grown at a restrictive temperature [14]. Deletion of *VPS74* in *cdc34-2* cells abrogates the elongated bud morphology [15]. Whether this phenotype is linked to the glycosyltransferase retention or retrograde transport functions of Vps74p and the requirement for Vps74p in other transport and polarity development pathways is unknown.

In addition to genetic analyses, biochemical and cell biological analyses of Vps74p and its mammalian homologues have identified potential roles for Vps74p *in vivo*. Vps74p is a member of a conserved PtdIns(4)P-binding protein family. These proteins localize at the Golgi and are thought to function in anterograde transport pathways. Vps74p interacts directly with the PtdIns(4)P phosphatase Sac1p [16]. This interaction promotes the dephosphorylation of PtdIns(4)P, resulting in a reduction in PtdIns(4)P at the medial Golgi and membrane lipid homeostasis maintenance. The drosophila homologue GOLPH3 functions at the Golgi by binding directly and specifically to Golgi membrane through PtdIns(4)P. Golgi GOLPH3 bridges PtdIns(4)P and actomyosin (MYO18A), stretching and shaping the Golgi to promote vesicle budding [17]. In addition, the two mammalian Vps74p orthologues, GOLPH3 and GOLPH3-like, are components of the Golgi matrix and provide a dynamic scaffold for cargo sorting and membrane transport [18,19]. GOLPH3 physically interacts with VPS35, a subunit of the retromer protein-recycling complex, and enhances signaling through the mammalian target of rapamycin (mTOR) [20,21]. Overexpression of GOLPH3 results in hyperactivation of the mTOR signaling pathway, which may lead to oncogenic transformation [20]. These data and the finding that the expression of human GOLPH3 is elevated in many tumor tissues suggest that GOLPH3 is a first-in-class Golgi oncoprotein [20]. Deletion of *VPS35* in budding yeast results in rapamycin hypersensitivity [22]. However, whether yeast Vps74p also participates in modulating yeast TOR signaling and rapamycin sensitivity remains to be elucidated.

Structural analyses have demonstrated the importance of the C-terminal region of Vps74p for its required tetramerization and PtdIns(4)P binding events during Golgi localization and glycosyltransferase retention [7,23]. However, whether this region is required for all putative Vps74p functions remains unknown. Several residues at the N-terminus of Vps74p have been reported to be phosphorylated based on phosphoproteome analyses using mass spectrometry [24]. This observation leads us to suspect that the N-terminal domain of Vps74p and its phosphorylation also plays a significant role in Vps74p function. In this study, we report that Vps74p uses different domains to modulate apical polarized growth, the retention of glycosyltransferases in the Golgi, and rapamycin hypersensitivity, suggesting that Vps74p differentially controls a variety of cellular functions.

## Results

### The N-terminus of Vps74p is phosphorylated

**Figure 1:**
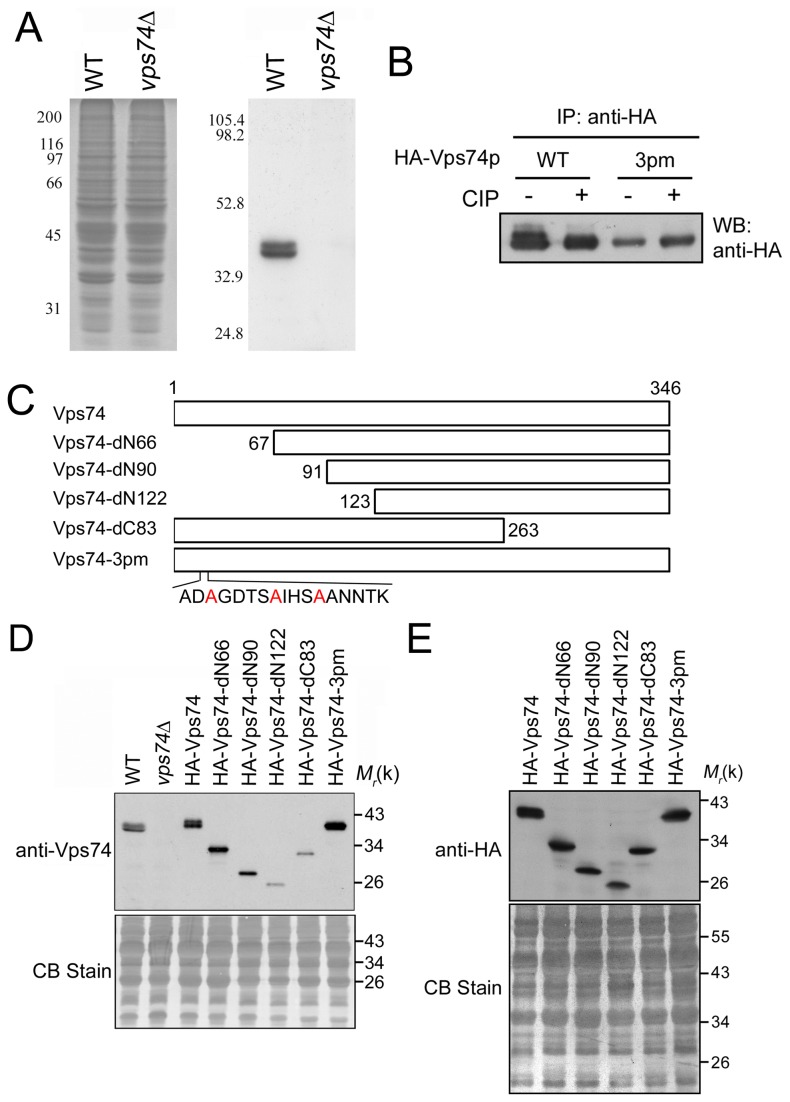
Vps74p is a phospho-protein. (A) The anti-Vps74p antibody is specific, Western blot analyses of crude lysates of wild-type (WT) and *vps74*Δ mutant yeast probed with antibodies against Vps74p (right) and SDS-PAGE stained with Coomassie Blue (left) demonstrates the specificity of the Vps74p antibody. (B) Cell lysis and immunoprecipitation assays were performed as described in the Materials and methods section. Immunoprecipitated proteins were separated into two equal portions and incubated at 37°C in CIP buffer for 1 h in the absence or presence of CIP. Each sample was subjected to SDS-PAGE followed by Western blotting using anti-HA antibody. (C) Schematic representation of the structure of the 346-residue Vps74p polypeptide and various mutant constructs is illustrated. (D) and (E) Expression of wild-type and mutant proteins is dipicted. HA epitope-tagged wild-type (WT) Vps74p, Vps74p-dN66, Vps74p-dN90, Vps74p-dN122, Vps74p-dC83, and Vps74p-3pm were expressed from 2μ-based plasmids (pVT101U) under the control of the *ADH* promoter in BY4741 yeast strain and detected by western blotting using the indicated antibodies.

To characterize the function of Vps74p, we first generated an antibody against Vps74p. Western blot analysis of wild-type yeast total cell lysates using our anti-Vps74p antibody showed two distinct bands that migrated closely (~39 to 41 kDa, the expected size for Vps74p); however, these bands were absent in lysates obtained from *vps74*Δ cells (Figure 1A). Vps74p has been shown to have at least three phosphorylation sites at serines 14, 19, and 23 [24]. To assess whether these two bands in the Western blot analysis represented the phosphorylated and non-phosphorylated forms of Vps74p, we used an anti-HA antibody to perform immunoprecipitation using lysates from yeast expressing HA-tagged Vps74P (WT) and calf intestine alkaline phosphatase (CIP)-treated immunoprecipitated HA-tagged Vps74p. As shown in Figure 1B, two bands were detected by Western blot analysis after immunoprecipitation. However, after CIP treatment, the lower mobility band disappeared. This suggests that the low mobility (upper) band might be a phosphorylated form of Vps74p.

To evaluate the significance of Vps74p S14, S19, and S23 phosphorylation *in vivo*, we constructed a HA-tagged triple point mutation of Vps74p (HA-Vps74p-3pm) that could not be phosphorylated at residues -14, -19, and -23 by replacing these serines with alanines. When this mutant was expressed in yeast using a high copy number plasmid, HA-Vps74p-3pm appeared to migrate as a single band; however, we observed two distinct bands representing the overexpressed wild-type HA-Vps74p. The mobility of the HA-Vps74p-3pm matched that of the higher mobility band of HA-Vps74p. We also evaluated CIP-treated immunoprecipitated HA-Vps74p-3pm and found that Vps74p-3pm produced only the lower band, regardless of whether the samples were subjected to CIP treatment (Figure 1B). These results confirmed that Vps74p is a phospho-protein, and this non-phosphorylatable Vps74p construct was used in further studies of Vps74p to characterize the function of Vps74p in yeast.

Structural analyses of Vps74p have indicated that Vps74p might form tetramers and that oligomerization is required for its Golgi localization [7]. Several regions within Vps74p contribute to oligomerization of the protein: α-helixes 1, 2, 9, and 10 and β-hairpins 3 and 4 [7]. To further characterize Vps74p functions in yeast, we constructed several truncated mutants of Vps74p. We truncated the N-terminal 66 (truncated before α-1), 90 (α1-deleted), or 122 (α1- and α2-deleted) amino acids or the C-terminal 83 amino acids (α9 through 12 deleted) (Figure 1C). Upon exogenous overexpression in yeast, overexpressed HA-Vps74p migrated as two distinct bands. As shown in Figure 1D, the three N-terminally truncated Vps74p mutants, HA-Vps74p-dN66, -dN90, and -dN122, migrated as one band when detected with anti-Vps74p and anti-HA antibodies, supporting the notion that Vps74p was phosphorylated at the N-terminus. HA-Vps74p-dC83 also migrated as one band, suggesting that the C-terminal deletion might affect the phosphorylation at the N-terminus of the protein.

### The *N-*terminal domain (1–66) is dispensable for Vps74p localization to the Golgi

**Figure 2:**
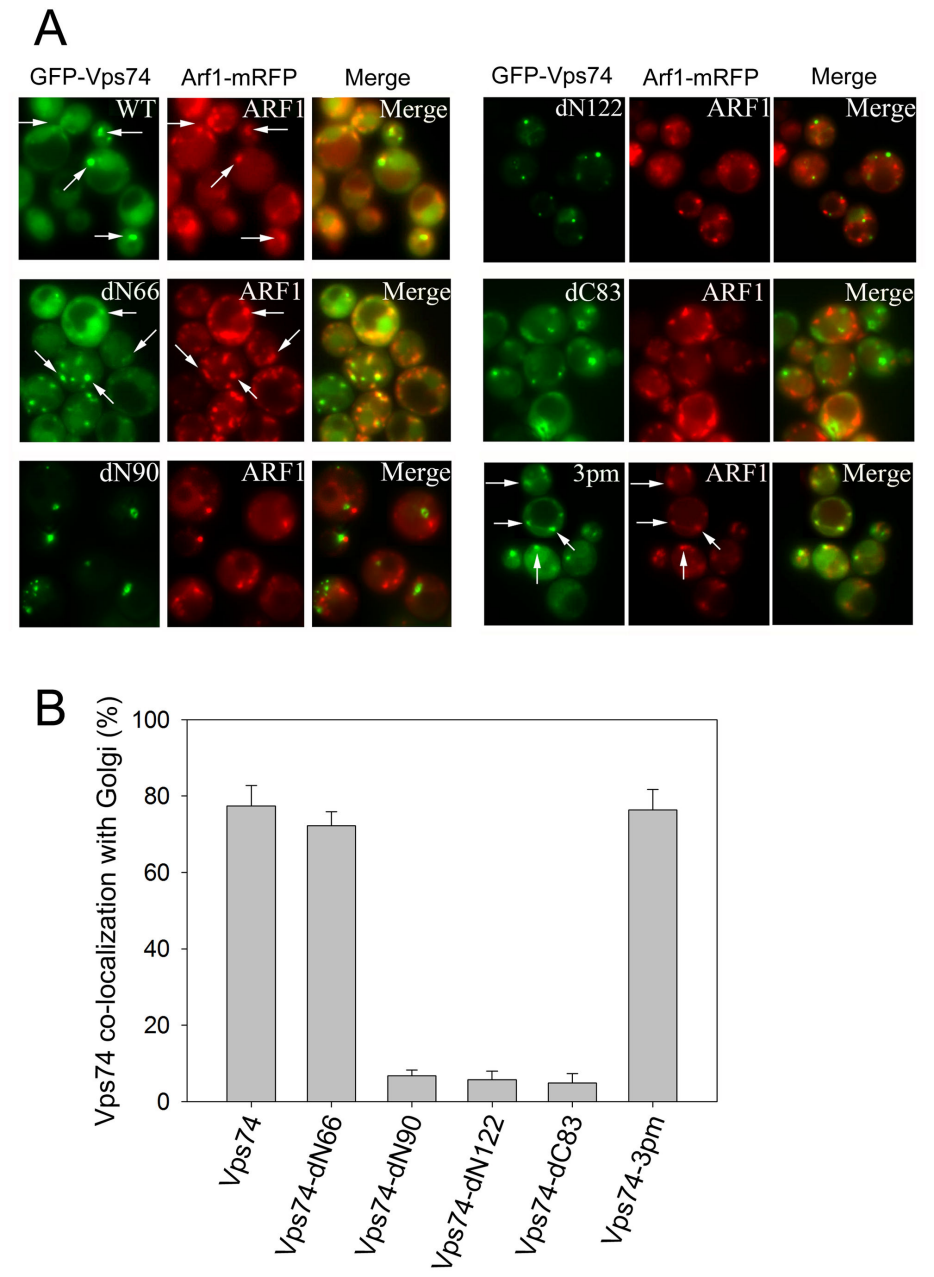
Vps74p is localized to the Golgi apparatus. N-terminal GFP-fused-Vps74p, -Vps74p-dN66, -Vps74p-dN90, -Vps74p-dN122, -Vps74p-dC83, and -Vps74p-3pm from a 2μ vector (pVT101U) under an *ADH* promoter were transformed into *vps74*-deleted yeast cells containing Arf1p-mRFP. Mid-log phase cells were imaged live via microscopy. (B) The quantitative measurements of the Golgi co-localization are presented.

To examine whether phosphorylation is required for Vps74p localization to the Golgi and to identify the sequence elements that contribute this event, we examined the localization of non-phosphorylatable and truncated mutants of Vps74p. Wild-type and mutant forms of Vps74p were tagged with GFP at their N-termini and each of these constructs were co-transformed into a *vps74*Δ mutant yeast strains with either Arf1p-mRFP or Arl1p-mRFP, (Golgi markers that reside in the *cis*- or trans-Golgi, respectively) (Figure 2). Wild-type Vps74p partially co-localized with Arf1p and Arl1p (Figures 2 and S1), indicating that Vps74p localizes to both the *cis*- and trans-Golgi networks. Vps74p-dN66 and Vps74p-3pm, also exhibited partial co-localization with Arf1p and Arl1p, indicating that neither phosphorylation nor the N-terminal 66 amino acids are required for Golgi localization of Vps74p. However, GFP-tagged Vps74p-dC83, Vps74p-dN90, and Vps74p-dN122 signals were observed on some punctuate structures that did not co-localize with the Golgi markers. These findings clearly indicated that the N-terminal 66 amino acids are not required for the association of Vps74p with the Golgi.

### The *N-*terminal 66 residues of Vps74p are required for its function on glycosyltransferase retention and Gas1p processing

**Figure 3:**
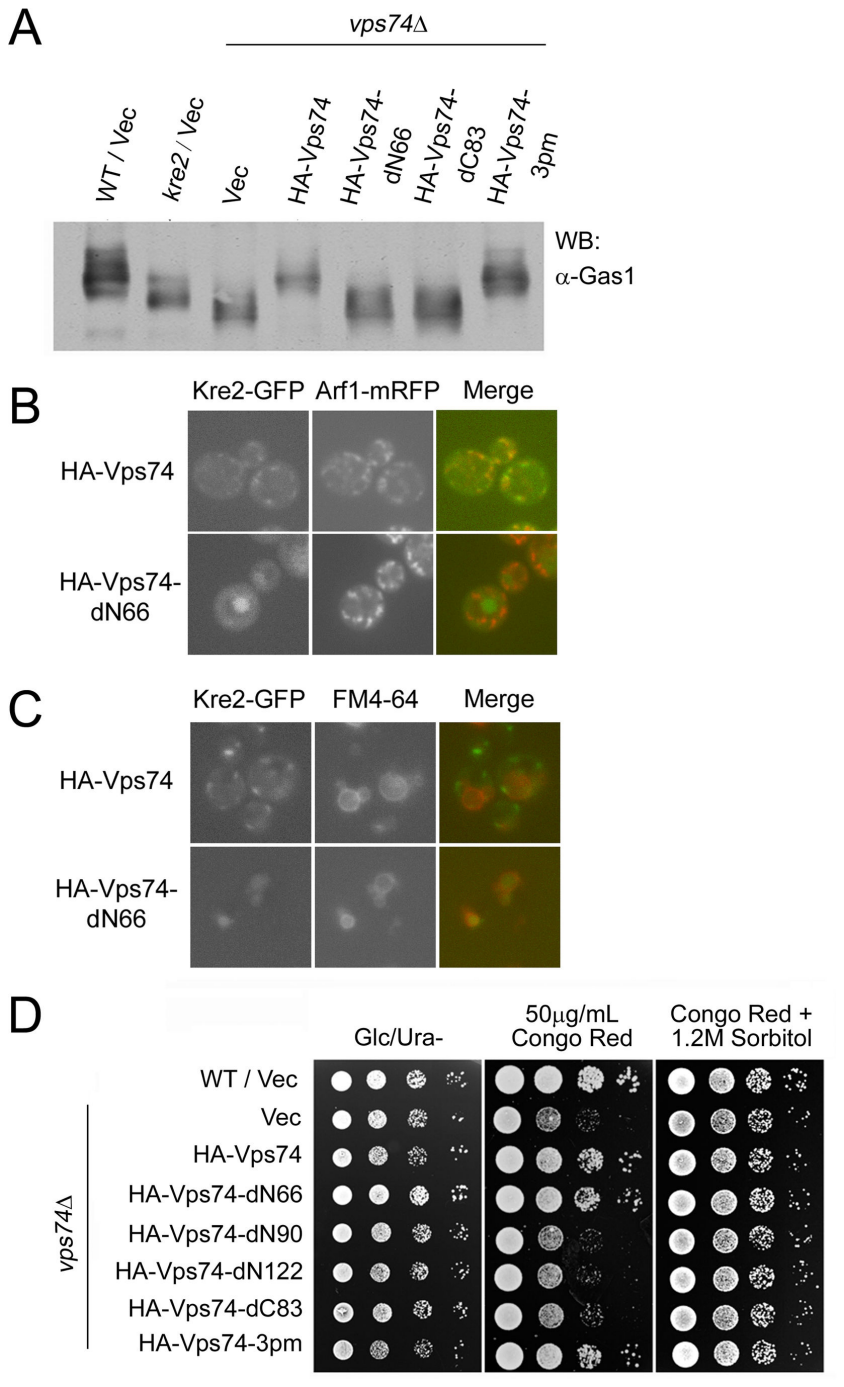
The effects of Vps74p on cell wall integrity are not due to the mislocalization of glycosyltransferases. (A) BY4741 wild-type, *kre2*Δ, and *vps74*Δ mutants were transformed with the empty vector pVT101U (Vec). BY4741 *vps74*Δ was transformed with HA-tagged Vps74, Vps74p-dN66, Vps74p-dC83, and Vps74p-3pm. Lysates prepared from these cells were subjected to Western blot analyses using an anti-Gas1p antibody. (B) and (C) The localization of Kre2p-GFP in *vps74*Δ mutant cells. HA-VPS74 and HA-VPS74-dN66, under the control of the *ADH* promoter in a 2μ plasmid, was transformed into *vps74*Δ mutant strains expressing Kre2p-GFP from its native locus. Transformants were grown in selection medium and the GFP signals were observed. Expression of Arf1p-mRFP was used as a Golgi marker. FM4-64 tracer dye was used for vacuole localization. (D) BY4741 was transformed with the empty vector pVT101U (Vec), and BY4741 *vps74*Δ yeast were transformed with pVT101U (Vec) and HA-tagged Vps74p, Vps74p-dN66, Vps74p-dN90, Vps74p-dN122, Vps74p-dC83, and Vps74p-3pm. These cells were cultured to mid-log phase. Subsequently, 10-fold serial dilutions were spotted onto Ura minus plates containing 2% glucose (left panel), YPD plates containing 50 µg/ml Congo red (middle panel), or YPD plates containing 50 µg/ml Congo red and 1.2 M sorbitol (right panel).

One suggested functions of Vps74p is to maintain the proper localization of Golgi-resident mannosyltransferases. Mislocalization of Golgi mannosyltransferases would affect the modification and processing of many cellular proteins, similar to the effect of deleting the mannosyltransferases themselves. Deletion of *KRE2* results in the hypoglycosylation of Gas1p, a plasma membrane β-1,3-glucanosyltransferase that is important for cell wall integrity [25,26]. Deletion of *VPS74* or disruption of Vps74p tetramerization and Golgi localization in yeast also results in the under-modification of Gas1p [6]. Therefore, we examined whether the N-terminal 66 amino acids of Vps74p are essential for Gas1p processing. As shown in Figure 3A, cells lacking *VPS74* displayed Gas1p processing defects. This activity was restored after overexpressing either full-length HA-Vps74p or the phosphorylation mutant HA-Vps74p-3pm. Overexpression of HA-Vps74p-dN66 or Vps74p-dC83 could not restore Gas1p processing in *vps74*Δ cells. These results indicate that both N- and C-terminal regions of Vps74p are required for Gas1p processing, however, the phosphorylation of Vps74p at the N terminus is not.

Vps74p is involved in the specific retention of mannosyltransferases, such as Kre2p, at the *cis*- and *medial*-Golgi compartments by binding directly to their cytoplasmic tails. In *vps74* mutant cells, Kre2p proteins are mislocalized to the vacuolar lumen [7]. We therefore examined whether the N-terminal 66 residues are required for the retention of Kre2p in the Golgi by assaying Kre2-mRFP in *vps74*Δ mutants expressing HA-Vps74p or HA-Vps74p-dN66. In *vps74*Δ cells overexpressing Vps74p, normal Golgi localization of Kre2p-mRFP was observed. However, in *vps74*Δ cells expressing Vps74p-dN66, Kre2p-mRFP did not localize to the Golgi (Figure 3B). These data indicated that the N-terminal 66-amino acid region of Vps74p is required for the retention of Kre2p in the Golgi. These results also suggested that Golgi localization alone is not sufficient for Vps74p to support the retention of Golgi mannosyltransferase and that different domains work in concert to execute the cellular function of Vps74p.

Deletion of specific genes involved in glycosylation events in yeast results in cell wall integrity defects and reduced viability in the presence of Calcofluor white (CW) or Congo red. Deletion of *VPS74* also resulted in Calcofluor white sensitivity, most likely due to the mislocalization of glycosyltransferases and the subsequent under-modification of proteins involved in the cell wall integrity in *vps74*Δ mutant cells [6]. To determine if Vps74p mutants that are unable to rescue Kre2p mislocalization or Gas1p under-modification phenotypes are also unable to rescue the cell wall integrity defects in *vps74*Δ cells, we examined the viability of *vps74*Δ cells and *vps74*Δ cells expressing various Vps74p mutants in the presence of Congo red. As shown in Figure 3C, *vps74*Δ mutants were hypersensitive to Congo red and this hypersensitivity could be rescued by the addition of 1.2 M sorbitol to adjust the osmolarity. This finding confirmed that the *vps74*Δ mutant hypersensitivity to Congo red results from defects in cell wall integrity. Moreover, none of the Vps74p-truncated mutants that failed to localize to the Golgi (-dN90, -dN122, or -dC83) repressed the Congo red hypersensitivity of *vps74*Δ cells, although wild-type and the -3pm mutant proteins did localize to the Golgi. Unexpectedly, the hypersensitivity was also repressed when we overexpressed the dN66-truncated mutant of Vps74p, despite the inability of this mutant protein to facilitate proper Gas1p glycosylation. Thus, under-processed glycosylated proteins cannot account for the cell wall integrity defects in *vps74*Δ mutant cells (Figure 3D), suggesting that Vps74p might be responsible for other unidentified cellular processes involved in maintaining the cell wall integrity.

### The *N-*terminal 66 residues of Vps74p are not required for cdc34-2-dependent apical growth

**Figure 4:**
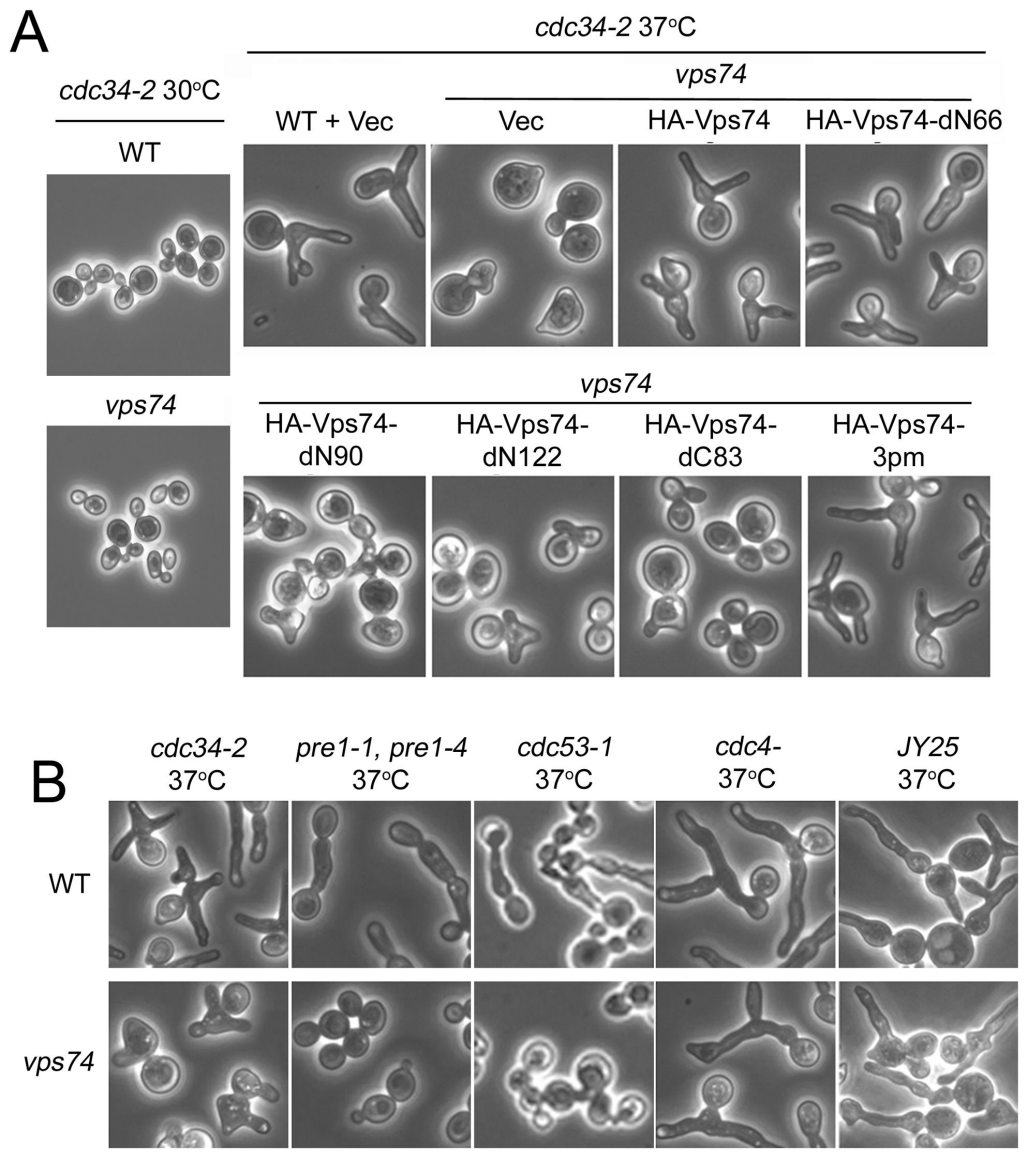
The N-terminal 66 residues of Vps74p are not required for *cdc34-2*-dependent apical growth. (A) Morphology transformants carrying the plasmid pVT101U (Vec) in *cdc34-2* mutant were detected. Transformed cells were grown in a synthetic selection medium at 25°C for 2 h and then shifted to 37°C for 6 h. Next, the ability to complement the elongating morphology was investigated using microscopy. Cells of *cdc34-2* with *VPS74* deleted were transformed with the indicated plasmids and then imaged as previously described. (B) Yeast strains *cdc34-2*, *pre1-1/pre4-1*, *cdc53-1*, *cdc4*-, *JY25*, *cdc34-2/vps74*Δ*, pre1-1/pre4-1/vps74*Δ*, cdc53-1/vps74*Δ*, cdc4-/vps74*Δ, and *JY25/vps74*Δ were grown to mid-log phase and these cultures were then transferred from room temperature to 37°C for 6 h and fixed at 37°C. The morphologies of these cells were visualized using microscopy.

Genetic screens for genes involved in abnormal apical growth have identified *VPS74* as one of the potential players in this process. Mutations in ubiquitin-dependent protein degradation pathways in yeast have been shown to induce abnormal apical growth, resulting in an elongated cell morphology [14,27,28]. Yeast cells harboring the temperature-sensitive *CDC34* allele (*cdc34-2*), which contains a mutation in an E2 ubiquitin-conjugating enzyme gene, cannot degrade G1 cyclins or a G2 cyclin/cdk inhibitor at a restrictive temperature [11,13,29]. Under the restrictive temperature, these cells remain at a stage of constitutive apical growth, leading to the formation of multiple highly elongated buds [30]. Deletion of *VPS74* in *cdc34-2* cells has been shown to reduce the formation of elongated buds [4]. Therefore, we examined whether specific domains in Vps74p are required to alter the elongated bud formation of *cdc34-2* cells at a restrictive temperature. Full-length *VPS74*, truncated constructs, and Vps74p-3pm were transformed into *cdc34-2/vps74*Δ mutants. As shown in Figure 4A, the buds in *cdc34-2* cells exhibited an elongated cell morphology. Deletion of the *VPS74* gene in *cdc34-2* cells reversed this phenotype, and the buds retained their round shapes. Overexpressing wild-type Vps74p in *cdc34-2/vps74*Δ cells restored the elongated bud formation observed in the *cdc34-2* mutant, indicating that Vps74p contributes to the formation of enlarged buds in *cdc34-2* cells. Upon overexpression of the different truncation and phosphorylation mutants in *cdc34-2/vps74*Δ cells, only cells expressing Vps74p-3pm or Vps74p-dN66 demonstrated elongated bud formation at a restrictive temperature. Cells expressing Vps74p-dN90, -dN122, or -dC83 did not exhibit elongated bud formation. These results indicate that Golgi-associated Vps74p-dN66 might retain certain aspects of Vps74p function that are required for apical growth. Our findings also indicate that phosphorylation of Vps74p at the three N-terminal serine sites is not required for this function.

### Vps74p is specifically involved in *cdc34*-dependent polarized apical growth

Yeast with mutations in several other proteins involved in cell cycle progression also form elongated buds. For example, mutations in ubiquitin-dependent protein degradation pathways (*cdc34*, *cdc53*, and *pre1-1/pre4-1*) in yeast induce abnormal apical growth, resulting in elongated cell morphologies [28]. Mutations in *CDC4*, an F-box protein required for G1/S and G2/M transitions, lead to the formation of elongated buds [14]. In addition, the great majority of *clbl,2,3,4* mutant cells (*JY25* yeast strains) arrest in S phase and show a cellular elongation defect at non-permissive temperatures [8,31]. To evaluate whether *VPS74* also interacts with these genes during elongated bud formation, we disrupted the *VPS74* gene in *pre1-1/pre4-1*, *cdc53-1*, *cdc4*- and *JY25* (*clb1*::*URA3*, *clb2* ^*ts*^, *clb3*::*TRP1*, *clb4*::*HIS3*) yeast strains. As shown in Figure 4B, when compared to the control parental strains, *pre1-1/pre4-1/vps74*Δ and *cdc53-1*/*vps74*Δ mutants exhibited a decreased degree of elongated bud formation. In contrast, the majority of the *cdc4/vps74*Δ and *JY25*/*vps74*Δ cells continued to form elongated buds. These results suggest that Vps74p is specifically involved in *cdc34*-dependent polarized apical growth.

We next examined whether Vps74p contributes to elongated bud formation via its ability to retain glycosyltransferases at the Golgi, which assures proper glycosylation of cell wall proteins. We deleted two glycosyltransferase genes, *KRE2* and *MNN1*, and the cell wall protein *GAS1* in *cdc34-2*, *cdc4*, or *JY25* cells and evaluated if the formation of elongated buds was altered. At non-permissive temperatures, *kre2*Δ and *mnn1*Δ mutations did not alter elongated bud formation in any of these cells (Figure S2). Because Gas1p is under-glycosylated in *vps74*Δ cells (Figure 3A), we speculated that the *gas1*Δ mutation might specifically alter elongated bud formation in *cdc34-2* cells, similar to *vps74*Δ. However, *GAS1* deletion reversed the elongated bud formation phenotype in all three mutant yeasts strains examined, indicating that Gas1p-mediated cell wall biosynthesis broadly affects apical growth elongation. Thus, these data indicate that the mislocalization of glycosyltransferases or under-modification of cell wall proteins, such as Gas1p, may not be the primary causes of *vps74*Δ mutation-dependent morphological changes, indicating that other putative functions of Vps74p might be involved in these processes.

### The PtdIns(4)P phosphatase Sac1p is involved in cdc34-2-dependent abnormal apical growth

**Figure 5:**
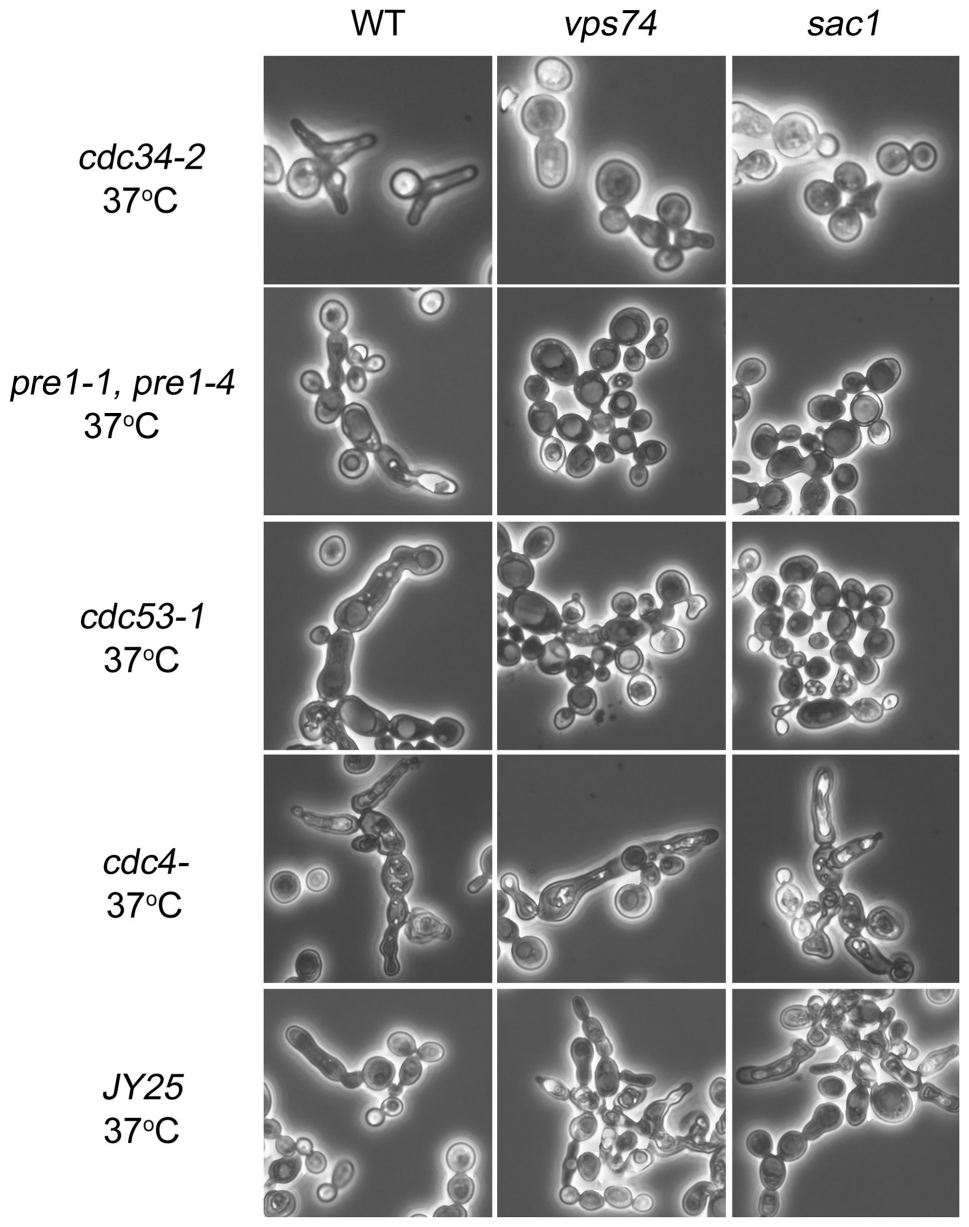
Sac1p is involved in *cdc34-2*-dependent apical growth. Yeast strains *cdc34-2, pre1-1 pre4-1, cdc53-1, cdc4-, JY25, cdc34-2/vps74*Δ*, pre1-1 pre4-1/vps74*Δ*, cdc53-1/vps74*Δ*, cdc4-/vps74*Δ*, JY25/vps74*Δ*, cdc34-2/sac1*Δ*, pre1-1 pre4-1/sac1*Δ*, cdc53-1/sac1*Δ*, cdc4-/sac1*Δ, and *JY25/sac1*Δ were grown to mid-log phase and then transferred to 37^o^C for 6 h. The morphologies of these cells were visualized using microscopy.

Vps74p can modulate Golgi PtdIns(4)P homeostasis via interaction with the PtdIns(4)P phosphatase Sac1 [16]. Sac1p has been implicated in the coordination of cytoskeletal and secretory activities, and *SAC1* deletion leads to defects in the cell wall integrity pathway [32]. To evaluate whether this aspect of Vps74p function contributes to elongated bud formation, we tested whether deletion of *SAC1* affects elongated bud formation in *cdc34-2* cells. At non-permissive temperatures, *cdc34-2/sac1*Δ, *pre1-1/pre4-1/sac1*Δ, and *cdc53-1*/*sac1*Δ cells did not form elongated buds, similar to *cdc34-2/vps74*Δ cells (Figure 5). In addition, *SAC1* deletion did not affect the apical growth of *cdc4*- and *JY25* mutants or Gas1p processing (data not shown). These results suggest that Sac1p is specifically involved in Cdc34p-regulated apical growth but does not retain glycosyltransferases at the Golgi.

### Arf1p participates in the abnormal apical growth of the cdc34-2 mutant

**Figure 6:**
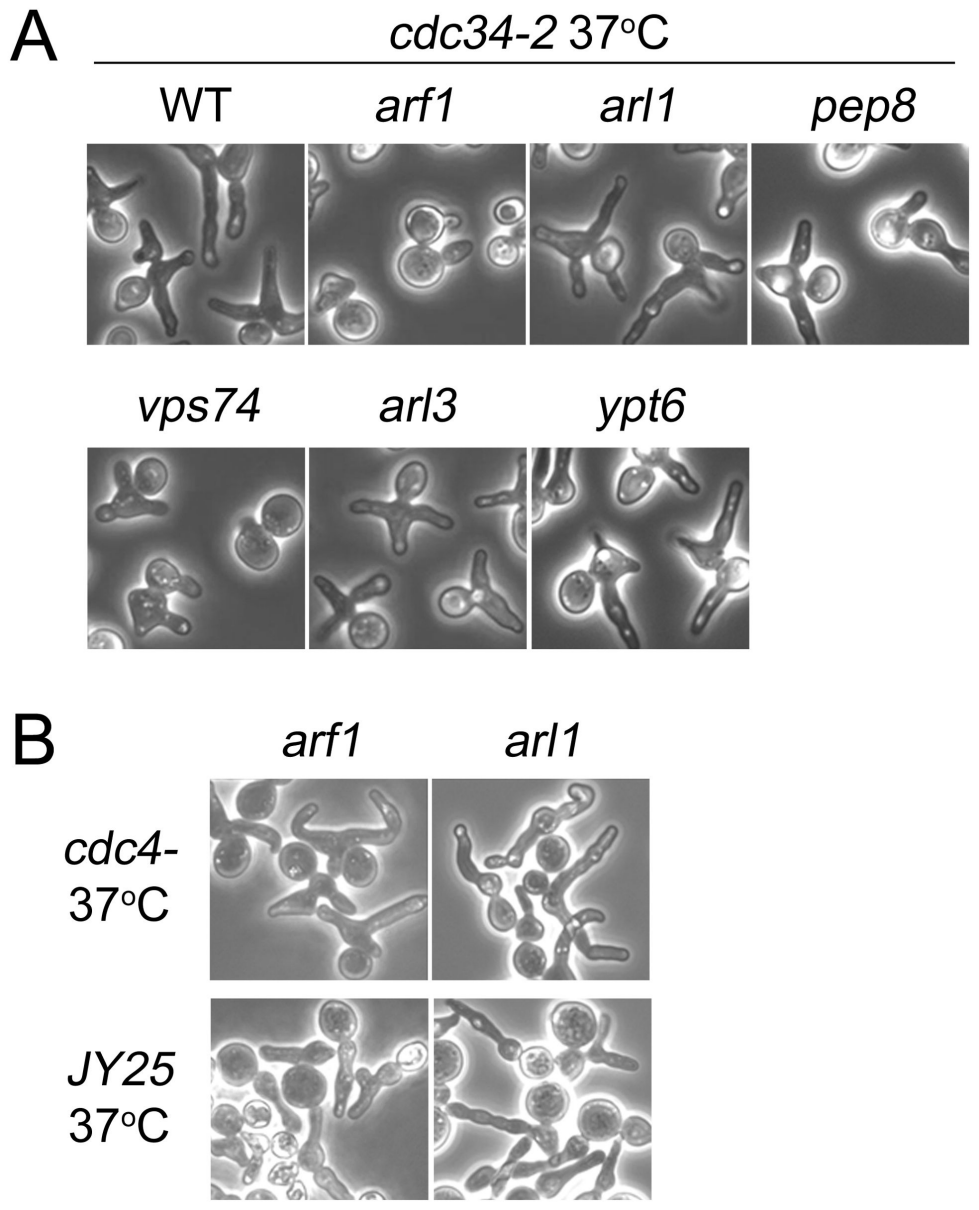
Arf1p facilitates elongated bud formation in *cdc34-2* mutant. (A) *cdc34-2, cdc34-2/vps74*Δ*, cdc34-2/arf1*Δ*, cdc34-2/arl1*Δ*, cdc34-2/arl3*Δ*, cdc34-2/pep8*Δ, and *cdc34-2/ypt6*Δ mutants (B) *cdc4-/arf1*Δ*, cdc34-2/arl1*Δ*, JY25/arf1*Δ*, and JY25/arl1*Δ mutants were grown in YPD at 25°C to an OD600 nm of 0.2, shifted to 37°C for 6 h, and imaged by microscopy.

Vps74p functions as an adaptor protein for COPI to recruit Golgi-resident glycosyltransferases into COPI-coated vesicles [6]. However, Vps74p-dN66, which cannot mediate Golgi glycosyltransferase recruitment into COPI-coated vesicles [33], reversed the phenotype of *cdc34-2/vps74*Δ cells (Figure 4A). We therefore examined whether the disruption of COPI vesicle formation affects apical growth in *cdc34-2* cells. The formation of COPI-coated vesicles is regulated by the small GTPase Arf1p. Arf1p is a member of ARF family of proteins that regulate coated vesicle formation. We examined whether deletion of *ARF1* in *cdc34-2* cells affects elongated bud formation. At a restrictive temperature, *cdc34-2/arf1*Δ cells did not form elongated buds, similar to *cdc34-2/vps74*Δ cells (Figure 6A). Two ARF family members, Arl1p and Arl3p, are known to regulate the specific vesicular transport pathway at the trans-Golgi. Therefore, we examined whether these two molecules also participated in the formation of elongated buds in *cdc34-2* cells. Neither Arl1p nor Arl3p were required for the elongated bud phenotype in *cdc34-2* cells (Figure 6A), indicating that Arf1p, but not Arl1p or Arl3p, is involved in *cdc34-2*-dependent elongated bud formation. To investigate whether Arf1p regulates *cdc34-2*-dependent apical growth, we also examined the morphology of *cdc4*- and *JY25* mutant cells containing an *arf1*Δ or *arl1*Δ mutation at non-permissive temperatures (Figure 6B). Neither the *arf1*Δ nor *arl1*Δ mutation affected apical growth in *cdc4*- and *JY25* mutants. *VPS74* has been shown to genetically interact with several proteins involved in retrograde transport pathways, including *PEP8* and *YPT6*. Pep8p is a vacuolar sorting protein that is essential for endosome-to-Golgi retrograde protein transport [34] and was identified as a Vps74p-binding partner in a comprehensive two-hybrid analysis [35]. Ypt6p is a Ras-like GTP-binding protein involved in the secretory pathway and is required for fusion of endosome-derived vesicles with the late Golgi [36,37] and has shown synthetic lethality with *VPS74* [3]. We therefore examined whether these molecules also contribute to abnormal bud formation in *cdc34-2* cells. Upon disruption of *PEP8 or YPT6* in *cdc34-2* mutants, the cells formed elongated buds at a restrictive temperature, indicating that the interaction of these genes with *VPS74* might not contribute to this phenotype (Figure 6A). Taken together, these results show that although the Vps74p-COPI interaction is not required for the role of Vps74p in mediating elongated bud formation, COPI vesicle formation or other Arf1p-dependent pathways are required for elongated bud formation in *cdc34-2* cells.

### Double deletion of VPS74 and ARF1 results in a synthetic cell wall integrity defect

**Figure 7:**
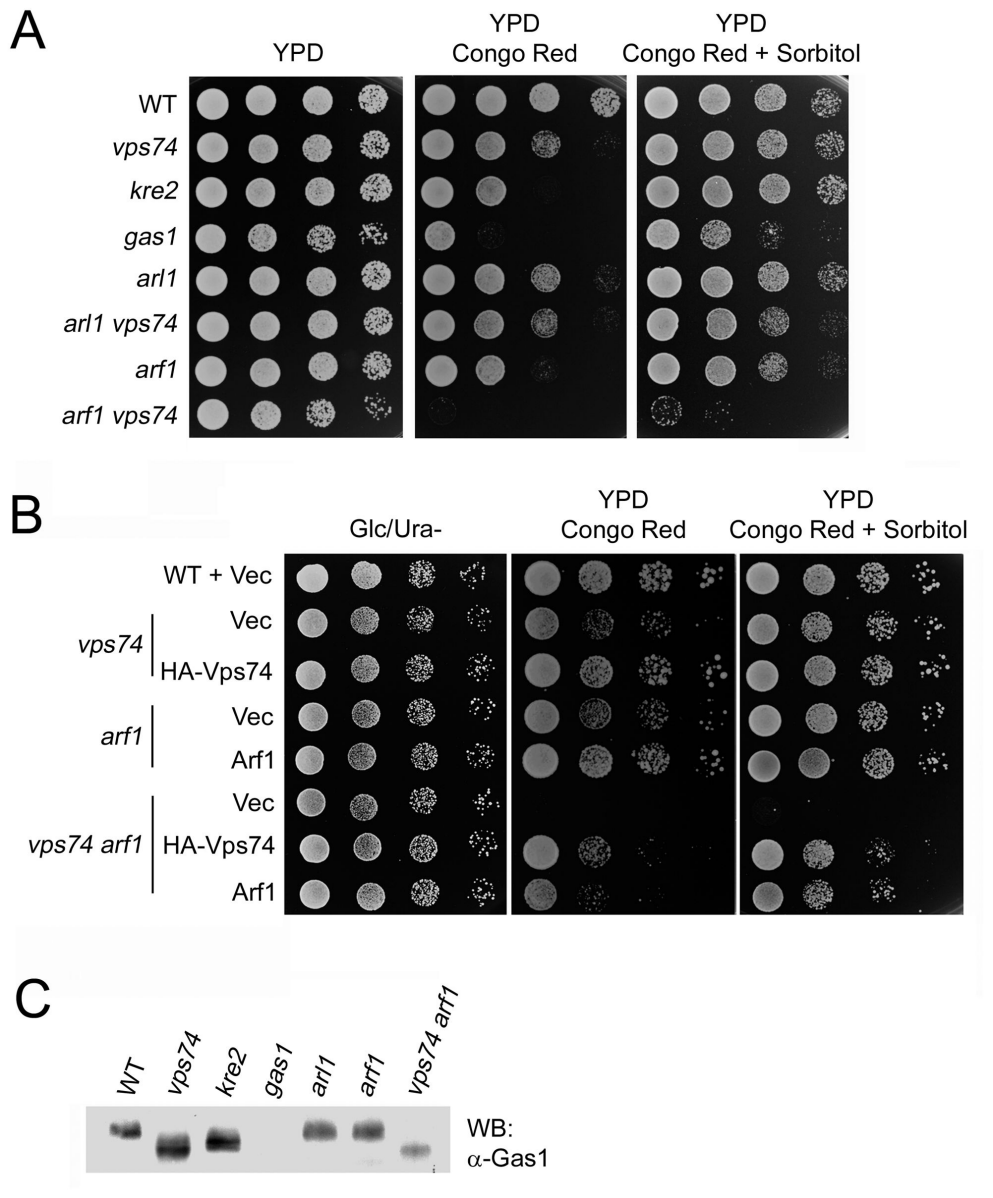
Deletion of *VPS74* in combination with *ARF1* results in a synergistic defect in cell wall integrity. (A) and (B) Indicated cells were cultured to mid-log phase, then 10-fold serial dilutions were spotted onto Ura minus plates containing 2% glucose (left panel), YPD plates containing 50 µg/ml Congo red (middle panel), or YPD plates containing 50 µg/ml Congo red and 1.2 M sorbitol (right panel). (C) Lysates were prepared from the indicated cells and proteins were precipitated with TCA to prepare an immunoblot that was probed with anti-Gas1p polyclonal antibodies.

*VPS74* and *ARF1* both modulate COPI-coated vesicle formation and similar phenotypes are observed upon deletion of either gene in *cdc34-2* cells. We therefore examined whether *ARF1* and *VPS74* functioned in concert or in parallel to facilitate these cellular processes. Arf1p is known to contribute to the maintenance of cell wall integrity. We first examined whether *vps74*Δ and *arf1*Δ double mutants were hypersensitive to Congo red. As shown in Figure 7A, similar to *kre2*Δ and *gas1*Δ mutants, both *vps74*Δ and *arf1*Δ mutants were sensitive to Congo red, and double deletion of *VPS74* and *ARF1* displayed a synthetic hypersensitivity to Congo red. This hypersensitivity could be partially rescued by expressing either Vps74p or Arf1p from low-copy *CEN* vectors, but not by osmolarity adjustment (addition of 1.2 M sorbitol), suggesting that the *vps74arf1*Δ double deletion mutant has a severe defect in cell wall integrity (Figure 7B). This result suggests that the function of Vps74p and Arf1p are related, in part, to different transport processes involved in cell wall integrity. Although deletion of another Golgi small GTPase, Arl1p, also resulted in Congo red sensitivity, the *arl1vps74*Δ double mutant did not exhibit a synthetic hypersensitivity to Congo red. We also examined whether Arf1p was required for Gas1p modification. Western blot analysis of Gas1p modification in *arf1*Δ cells revealed that, unlike *vps74*Δ cells, the Gas1p glycosylation was not affected in *arf1*Δ cells (Figure 7C). Taken together, these results indicated that Vps74p and Arf1p might function in parallel pathways contributing to apical growth and the maintenance of cell wall integrity.

### Phosphorylation of Vps74p contributes to rapamycin hypersensitivity

Recent studies have shown that overexpression of mammalian Vps74p and GOLPH3 results in cell transformation, tumor growth *in vivo*, and rapamycin hypersensitivity [20]. We examined the rapamycin sensitivity of yeast cells overexpressing Vps74p and found a similar effect: overexpression of full-length Vps74p resulted in rapamycin hypersensitivity. We next expressed various Vps74p truncated and non-phosphorylatable mutants to examine which region of Vps74p might be responsible for this phenotype. We found that none of the yeast cells expressing these mutants displayed rapamycin hypersensitivity, including Vps74p-3pm (Figure S3). These data suggested that the Golgi localization and phosphorylation of Vps74p contribute to rapamycin hypersensitivity. The effect of GOLPH3 on mTOR signaling has also been suggested to be mediated through the interaction of GOLPH3 with VPS35 to regulate retrograde transport and receptor recycling of key molecules [20]. We therefore examined the genetic interaction between *VPS74* and *VPS35* in yeast. High throughput screening data have shown that *VPS74* and *VPS35* both demonstrate synthetic lethality with *YPT6* deletion in yeast [3]. We overexpressed Vps74p in *vps35*Δ or *ypt6*Δ cells and found that overexpression of Vps74p did not enhance rapamycin hypersensitivity in either mutant cells (Figure S4). These results suggested that the rapamycin hypersensitivity observed upon Vps74p overexpression is dependent on Ypt6p- or Vps35p-modulated processes. Therefore, yeast Vps74p may play a similar role to that of mammalian VPS74-family proteins in inducing rapamycin hypersensitivity. Both the function of Vps74p in retrograde transport and phosphorylation at the N-terminal domain of the protein contribute to the rapamycin hypersensitivity.

## Discussion

In this report, we demonstrate that Vps74p not only participates in the retention of glycosyltransferases at the Golgi, but also plays a role in apical growth and rapamycin hypersensitivity in yeast. Using mutational analyses, we discovered that different domains are required for each Vps74p function, suggesting that Vps74p differentially controls a variety of cellular functions (summarized in Table 1).

**Table 1:** Summary of the phenotypes of Vps74p and the mutant proteins.

Phenotype	Vps74p					
	WT	-dN66	-dN90	-dN122	-dC83	-3pm
Protein pattern	Double bands	Single	Single	Single	Single	Single
Vps74p Golgi localization	+	+	-	-	-	+
Gas1p processing	+	-	NA	NA	-	+
Kre2p-GFP Golgi localization	+	-	NA	NA	NA	NA
Restore Congo red hypersensitivity in *vps74*Δ cells	+	+	-	-	-	+
Elongated bud formation in *cdc34-2*	+	+	-	-	-	+
Rapamycin hypersensitivity in wild type yeast cells	+	-	-	-	-	-

The domain structure of Vps74p and other Vps74p-family members can be roughly divided into an N-terminal unstructured region (approximately 40-60 amino acids) and folded C-terminal regions. Two elegant studies characterizing the role of Vps74p in Golgi glycosyltransferase retention have revealed that the C-terminal folded domain of Vps74p is crucial for its oligomerization, PtdIns(4)P and glycosyltransferase binding, and association with the Golgi [7]. In this study, we found that the N-terminal region, although not required for Golgi localization or glycosyltransferase binding, is necessary for the function of Vps74p in glycosyltransferase retention. While this manuscript was in preparation, Tu et al. reported that an N-terminal conserved arginine-motif in GOLPH3-family proteins is required for coatomer binding. These authors demonstrated that the N-terminal 66 amino acids of Vps74p are necessary and sufficient to mediate Vps74p-coatamer interaction. Furthermore, these authors found that three arginine residues (amino acids 6-8) are essential for coatomer binding of Vps74p [33]. Taken together, these results show that Vps74p provides a bridge for Golgi glycosyltransferase and vesicle coat proteins together, ensuring the packaging of these enzymes into retrograde transport vesicles.

The deletion of several genes involved in glycosylation, such as *KRE2*, results in cell wall integrity pathway defects, thus reducing the viability of yeast in the presence of Calcofluor white or Congo red. We and others have shown that *vps74*Δ mutant yeast are more sensitive to Calcofluor white or Congo red than *kre2*Δ cells. Tu et al. have reasoned that Vps74p could mediate the Golgi localization of multiple glycosyltransferases [6]. However, when we overexpressed Vps74p lacking the first 66 amino acids in a *vps74*Δ mutant, the viability of the resulting cells was improved to a level similar to that of cells expressing full-length, wild-type Vps74p. Localization of Golgi-glycosyltransferases was not rescued, and cell wall proteins such as Gas1p were under-modified in *vps74*Δ cells expressing dN66. These findings indicate that the dN66 form of Vps74p could not function to retain glycosyltransferases at the Golgi, nor did it rescue the glycosylation of cell wall proteins. Expression of the dN66 form of Vps74p also ameliorates the cell wall integrity defects, despite the failure of this mutant to retain glycosyltransferases at the Golgi. These data indicate that the cell wall integrity defects caused by the *VPS74* gene deletion were not merely a result of defects in multiple glycosyltransferase retention events and suggested that a novel function of Vps74p contributes to the maintenance of cell wall integrity.

The cell cycle-dependent switch between apical and isotropic growth contributes to the maintenance of the ellipsoidal shape of *S. cerevisiae*. We validated the large-scale screen result that deletion of *VPS74* in *cdc34-2* cells abrogates the prolonged apical growth phase at a restrictive temperature. Apical growth of yeast cells is heavily dependent on polarized vesicular transport. Given that Vps74p functions in cargo sorting in Golgi-derived COPI vesicles, the mechanism underlying the reversal of the prolonged apical growth may be related to the Golgi-localization and function of Vps74p. As expected, Vps74p mutants that are unable to bind to glycosyltransferases and PtdIns(4)P at the Golgi (namely, the C-terminal truncation mutant) could not restore elongated bud formation. However, the expression of Vps74p-dN66, a mutant that cannot bind to the coatomer [6] or facilitate glycosyltransferase retention, facilitated elongated bud formation in *cdc34-2/vps74*Δ double mutants at restrictive temperatures. Furthermore, *VPS74* genetically interacts with *YPT6* and *PEP8*, both of which are thought to be involved in vesicular transport. Deletion of these genes in the *cdc34-2* mutant did not phenocopy the *cdc34-2/vps74*Δ double mutant. Thus, the ability of Vps74p to modulate apical growth in *cdc34-2* cells at restrictive temperatures might not be related to its role in retrograde transport.

Among the genes examined, *ARF1* was the only gene involved in Golgi-related vesicular transport that could also modulate apical growth. Arf1p is an ARF family small GTP-binding protein that promotes the formation of COPI-coated vesicles at the Golgi. Deletion of the *ARF1* results in a defect in cell wall integrity and anterograde transport. Deletion of other ARF family proteins, such as *ARL1*, also leads to transport defects, glycoprotein under-modifications, and cell wall integrity defects. However, deletion of *ARL1* did not show an alteration in bud morphology in the *cdc34-2* mutant. These results suggested that the reversal of elongated bud formation in *cdc34-2/vps74*Δ or *cdc34-2/arf1*Δ cells is not a non-specific phenomenon resulting from disruption of a vesicular transport events. Our findings that Vps74p-truncated mutants that are unable to support glycosyltransferases retention could rescue the *cdc34-2/vps74*Δ phenotype and that *ARF1* and *VPS74* displayed a synthetic defect in cell wall integrity further confirm that the role of Vps74p extends beyond simply mediating the packaging of Golgi resident glycosyltransferases into COPI vesicles.

What are the other potential function(s) of Vps74p? Reports by Wood et al. have indicated that Vps74p might act as a sensor of PtdIns(4)P levels in the *medial*-Golgi [16]. These authors have shown that Vps74p interacts directly with Sac1p (a PtdIns(4)P phosphatase) and that this interaction promotes the dephosphorylation of PtdIns(4)P, thereby reducing the amount of PtdIns(4)P at the *medial*-Golgi and maintaining membrane lipid homeostasis. Perturbation of PtdIns(4)P homeostasis has been shown to induce cell wall integrity pathway defects. Deletion of the *SAC1* gene, similar to *vps74*Δ, caused Calcofluor white sensitivity. Deleting *SAC1* in *cdc34-2* cells resulted in an amelioration of the elongated bud phenotype in *cdc34-2* cells. Similar to the *VPS74* deletion, the effect of *SAC1* deletion on elongated bud formation was *cdc34-2* specific. Deletion of *SAC1* did not affect Gas1p processing, suggesting that Sac1p does not function in retaining glycosyltransferases at the Golgi. Thus, the suppression of *cdc34-2*-dependent apical growth induced by the *VPS74* deletion was due to a defect in PtdIns(4)P homeostasis and that N-terminal Vps74p deletion dN66, but not dN90, might have retained the ability to serve as a PtdIns(4)P sensor in Sac1p function. Previous studies have shown that PtdIns(4)P regulates the targeting of clathrin adaptor AP-1 complexes to the Golgi [38]. Thus, Vps74p may act with Sac1p on the TGN to support the export of a yet uncharacterized cargo protein for apical growth.

Previous studies on the mammalian orthologue of Vps74p, GOLPH3, have indicated that GOLPH3 overexpression contributes to rapamycin hypersensitivity in mammalian cells. In this report, we found that overexpression of Vps74p results in rapamycin hypersensitivity in yeast. Additionally, both the N-terminal unstructured domain and the C-terminal folded domain are required to induce rapamycin hypersensitivity. Interestingly, phosphorylation of Vps74p in the N-terminal domain is also required. Phospho-proteomic analysis has indicated that Vps74p is a phosphoprotein with three putative phosphorylation sites [39]. We confirmed that Vps74p is phosphorylated in vivo. However, using a mutant Vps74p that could not be phosphorylated revealed activities in glycosyltransferases retention, Congo red sensitivity, and apical growth when compared to wild-type Vps74p. Consistent with our findings, Tu et al. have also reported that mutations in two phosphorylation sites do not alter the ability of Vps74p to interact with coatomers. These results suggest that Vps74p is involved in the yeast TOR signaling pathway to modulate other aspects of yeast cell functions. Identification of upstream and downstream regulators that modulate Vps74p phosphorylation will help to further elucidate Vps74p function *in vivo* and provide a research platform to better understand the relationship between GOLPH3 activities and tumorigenesis in mammalian cells.

In this report, we unveiled many different aspects of Vps74p function. The interacting partners for Vps74p function include cell cycle regulators, signaling molecules and proteins involved in glycosylation, vesicular transport, and lipid homeostasis at the Golgi. Future studies characterizing Vps74 functions and molecular mechanisms underlying the modulation of Vps74p activity will provide additional insight into the basis for the coordination of multiple processes during the cellular life cycle.

## Materials and Methods

### Strains, media, and microbiological techniques

Table S1 lists the yeast strains used in this study. Yeast culture media were prepared as previously described [15]. YPD contained 1% Bacto-yeast extract, 2% Bacto-peptone, and 2% glucose. SD contained 0.2% Difco yeast nitrogen base (without amino acid), 0.5% ammonium persulfate, and 2% glucose. Nutrients essential for the auxotrophic strains were supplied at the specified concentrations. Yeast strains were transformed using the lithium acetate method [40]. Plasmids listed in Table S2 were constructed according to standard protocols. Gene disruption was performed as previously described [41].

### Preparation of yeast cell extracts and Western blot analyses

Whole yeast extracts were prepared by agitating yeast cells suspended in TE buffer (10 mM Tris, pH 7.4, 1 mM EDTA) with glass beads for 1 min followed by incubation on ice for 1 min, and this was repeated 5 times. After a brief centrifugation to clarify the lysate, the protein was quantified using the Coomassie blue assay (Pierce). Proteins separated by SDS-PAGE were transferred onto PVDF membranes (Millipore), which were incubated (60 min, room temperature) with antibodies in Tris-buffered saline (pH 7.4) containing 0.1% Tween 20 and 5% dried skim milk. Bound antibodies were detected using the ECL system (Amersham Pharmacia Biotech).

### Expression and purification of recombinant proteins and polyclonal antibody production

To create the His-tagged Vps47p, a DNA fragment containing the *VPS74* coding region was generated after amplifying yeast genomic DNA using sequence-specific primers. The PCR product was purified and ligated to the expression vector pET30a (Novagen), yielding pET30a/Vps74p. The His-tagged fusion protein was synthesized in BL21(DE3) *E. coli* and purified on a Ni^2+^-NTA resin (Qiagen, Chatsworth, CA) as previously described [42]. Denatured, purified recombinant Vps74p isolated from an SDS-PAGE gel was used as an antigen to raise polyclonal antibodies in rabbits, as described previously [42]. The polyclonal antibodies were diluted 1:5000 for Western blotting analyses.

### Immunoprecipitation and phosphatase treatment

Yeast cells were harvested by centrifugation and washed once with ddH_2_O, and broken by vortexing with glass beads. The total cell protein extracts were obtained by precipitation with 4% (vol/vol) trichloroacetic acid (TCA), washed twice by cold ddH_2_O, solubilized in buffer containing 50 mM Tris-HCl, pH 7.5, 150 mM NaCl, and 1% SDS, and then denatured in 95°C for 5 min. The soluble fraction was then collect by centrifugation at 4°C. The volume of soluble fraction was brought to 1 ml with buffer containing 50 mM Tris-HCl, pH 7.5, 150 mM NaCl, 0.1% Triton-X 100, 1 mM DTT, and protease inhibitor and incubate with anti-HA conjugated agarose beads for 2 h at 4°C on rotator. The anti-HA beads-immune complex was then pelleted by centrifugation, washed three times with dilution buffer, separated into two portions to incubate in CIP buffer in the presence or absence of calf intestinal phosphatase (CIP) for 1 h at 37°C on a rotator. The beads were then washed twice in dilution buffer, suspended in SDS-PAGE sample buffer, and boiled for 10 min prior to separation by SDS-PAGE and Western blotting analyses.

### Microscopy

The morphologies of living cells were observed after overnight culture to the mid-log phase. Yeast cells were grown at 25°C for 2 h and then at 37°C for 6 h. The ability to complement the elongating morphology was investigated using a Zeiss Axioskop microscope equipped with a Cool Snap FX camera.

## Supporting Information

Figure S1 **Vps74p is localized to the Golgi apparatus.** N-terminal GFP-Vps74p, -Vps74p-dN66, -Vps74p-dC83, and -Vps74p-3pm on a 2μ vector pVT101U under an *ADH* promoter were transformed into *vps74*-deleted yeast containing Arl1p-mRFP. Mid-log phase cells were live imaged by microscopy.
(TIF)

Figure S2 **Mnn1p and Kre2p do not participate in apical growth.** *cdc34-2/gas1*Δ*, cdc4-/gas1*Δ*, JY25/gas1*Δ*, cdc34-2/kre2*Δ*, cdc4-/kre2*Δ*, JY25/kre2*Δ, *cdc34-2/mnn1*Δ*, cdc4-/mnn1*Δ, and *JY25/mnn1*Δ were grown to mid-log phase and then transferred from room temperature to 37°C for 6 h and fixed at 37°C. The morphologies of these cells were visualized by microscopy. In each experiment, 100 cells were quantified and analyzed.
(TIF)

Figure S3 **Rapamycin hypersensitivity upon overexpression of Vps74p but not the mutants.** (A) BY4741 was transformed with the pVT101U vector, HA-tagged Vps74p, Vps74p-dN66, Vps74p-dN90, Vps74p-dN122, Vps74p-dC83, and Vps74p-3pm. These cells were cultured to mid-log phase. Subsequently, 10-fold serial dilutions were spotted onto a Ura minus plate containing 2% glucose without (left panel) and 100 nM rapamycin (right panel) (B) Cell extracts were prepared and analyzed by Western blot analysis with polyclonal rabbit anti-Vps74p antiserum and anti-Pgk1p antibodies. Pgk1p was used as a loading control.
(TIF)

Figure S4 **Deletion of *YPT6* and *VPS35* suppress rapamycin hypersensitivity upon overexpression of Vps74p.** Empty vector or Vps74p under an *ADH* promoter were transformed into wild type, *ypt6*Δ, or *vps35*Δ mutant cells. The transformants were serially diluted and spotted on plates of YPD with (right panel) and without (left panel) 100 nM rapamycin to examine their hypersensitivity.
(TIF)

Table S1 Yeast strains used in this study.
(DOC)

Table S2 **Plasmids used in this study.**
(DOC)
