# Supplementary figures and images for: The N-Terminus of Vps74p Is Essential for the Retention of Glycosyltransferases in the Golgi but Not for the Modulation of Apical Polarized Growth in Saccharomyces cerevisiae
Source: PLoS One. 2013 Sep 3;8(9):e74715. doi: 10.1371/journal.pone.0074715 (PMC3760917; doi:10.1371/journal.pone.0074715)

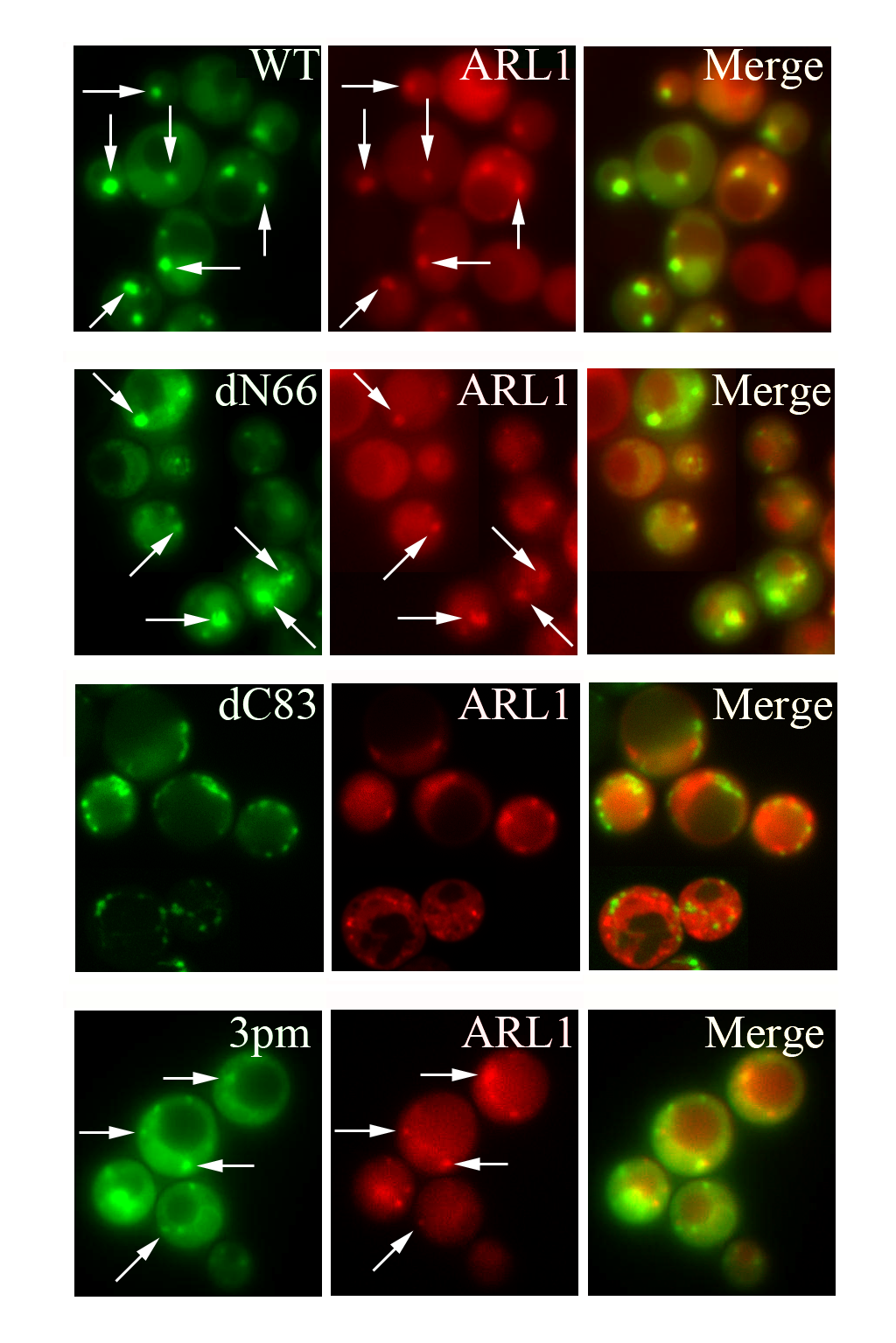

Supplement: Figure S1 — Vps74p is localized to the Golgi apparatus. N-terminal GFP-Vps74p, -Vps74p-dN66, -Vps74p-dC83, and -Vps74p-3pm on a 2μ vector pVT101U under an ADH promoter were transformed into vps74-deleted yeast containing Arl1p-mRFP. Mid-log phase cells were live imaged by microscopy. (TIF) [file pone.0074715.s001.tif]

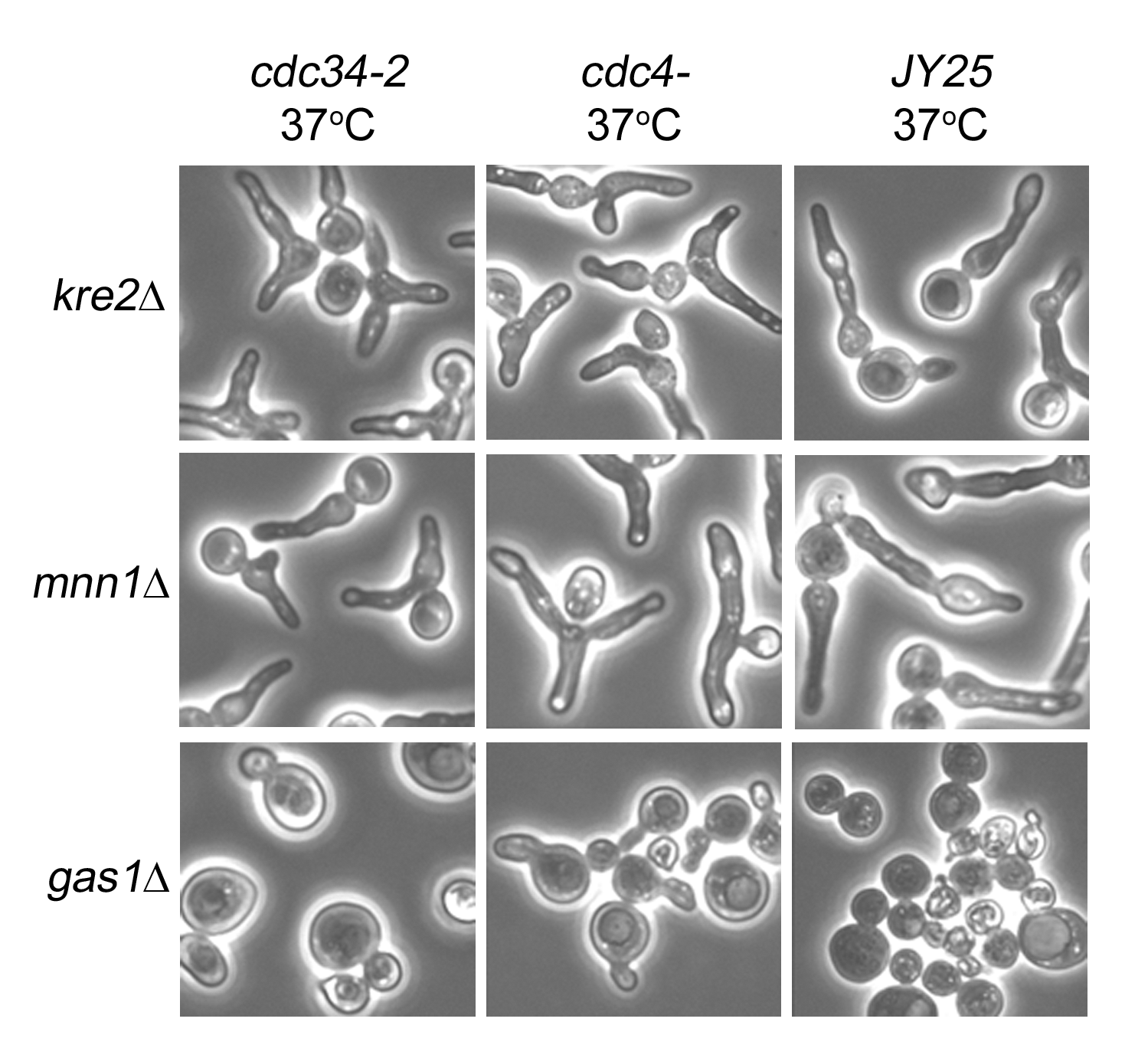

Supplement: Figure S2 — Mnn1p and Kre2p do not participate in apical growth. cdc34-2/gas1Δ, cdc4-/gas1Δ, JY25/gas1Δ, cdc34-2/kre2Δ, cdc4-/kre2Δ, JY25/kre2Δ, cdc34-2/mnn1Δ, cdc4-/mnn1Δ, and JY25/mnn1Δ were grown to mid-log phase and then transferred from room temperature to 37°C for 6 h and fixed at 37°C. The morphologies of these cells were visualized by microscopy. In each experiment, 100 cells were quantified and analyzed. (TIF) [file pone.0074715.s002.tif]

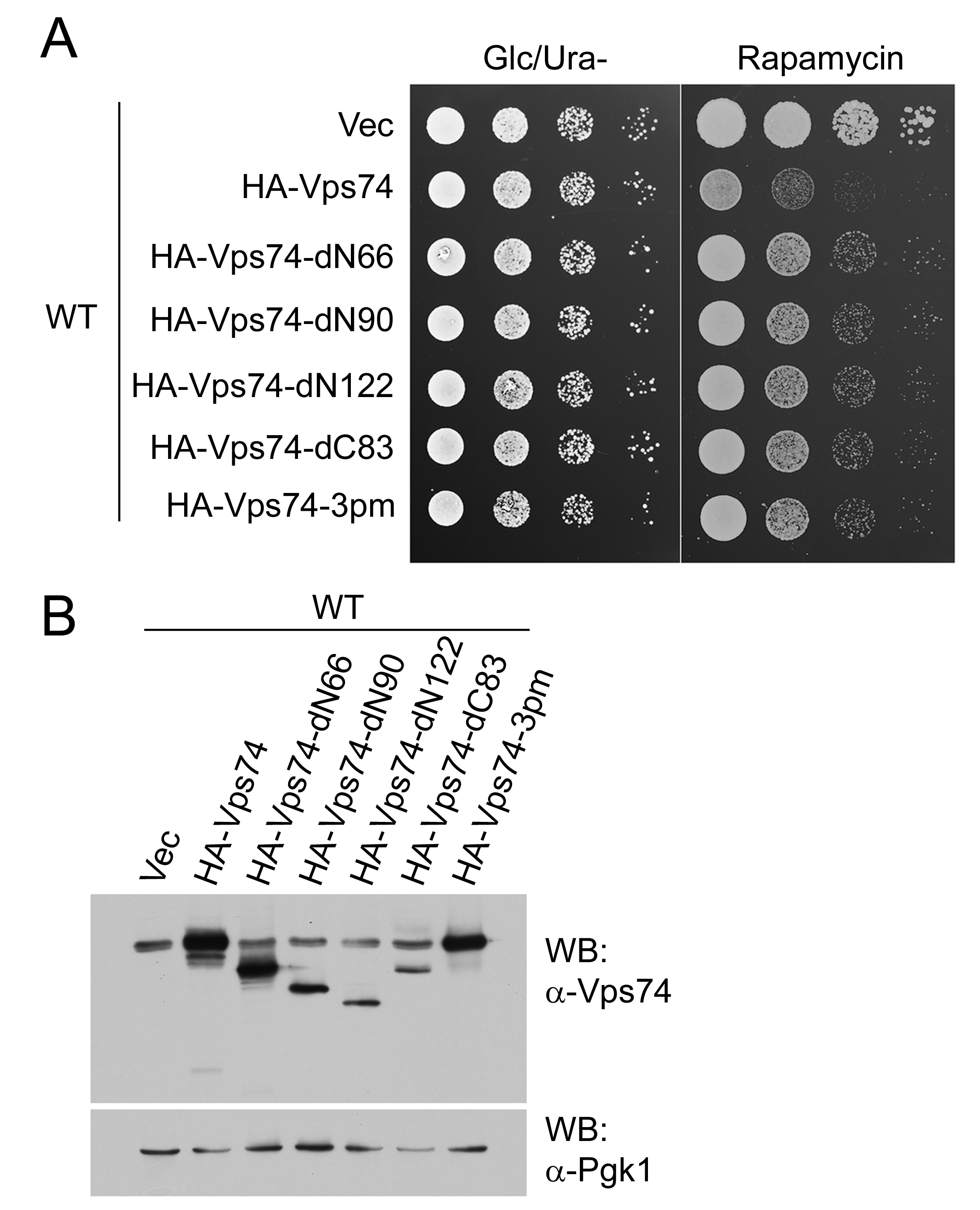

Supplement: Figure S3 — Rapamycin hypersensitivity upon overexpression of Vps74p but not the mutants. (A) BY4741 was transformed with the pVT101U vector, HA-tagged Vps74p, Vps74p-dN66, Vps74p-dN90, Vps74p-dN122, Vps74p-dC83, and Vps74p-3pm. These cells were cultured to mid-log phase. Subsequently, 10-fold serial dilutions were spotted onto a Ura minus plate containing 2% glucose without (left panel) and 100 nM rapamycin (right panel) (B) Cell extracts were prepared and analyzed by Western blot analysis with polyclonal rabbit anti-Vps74p antiserum and anti-Pgk1p antibodies. Pgk1p was used as a loading control. (TIF) [file pone.0074715.s003.tif]

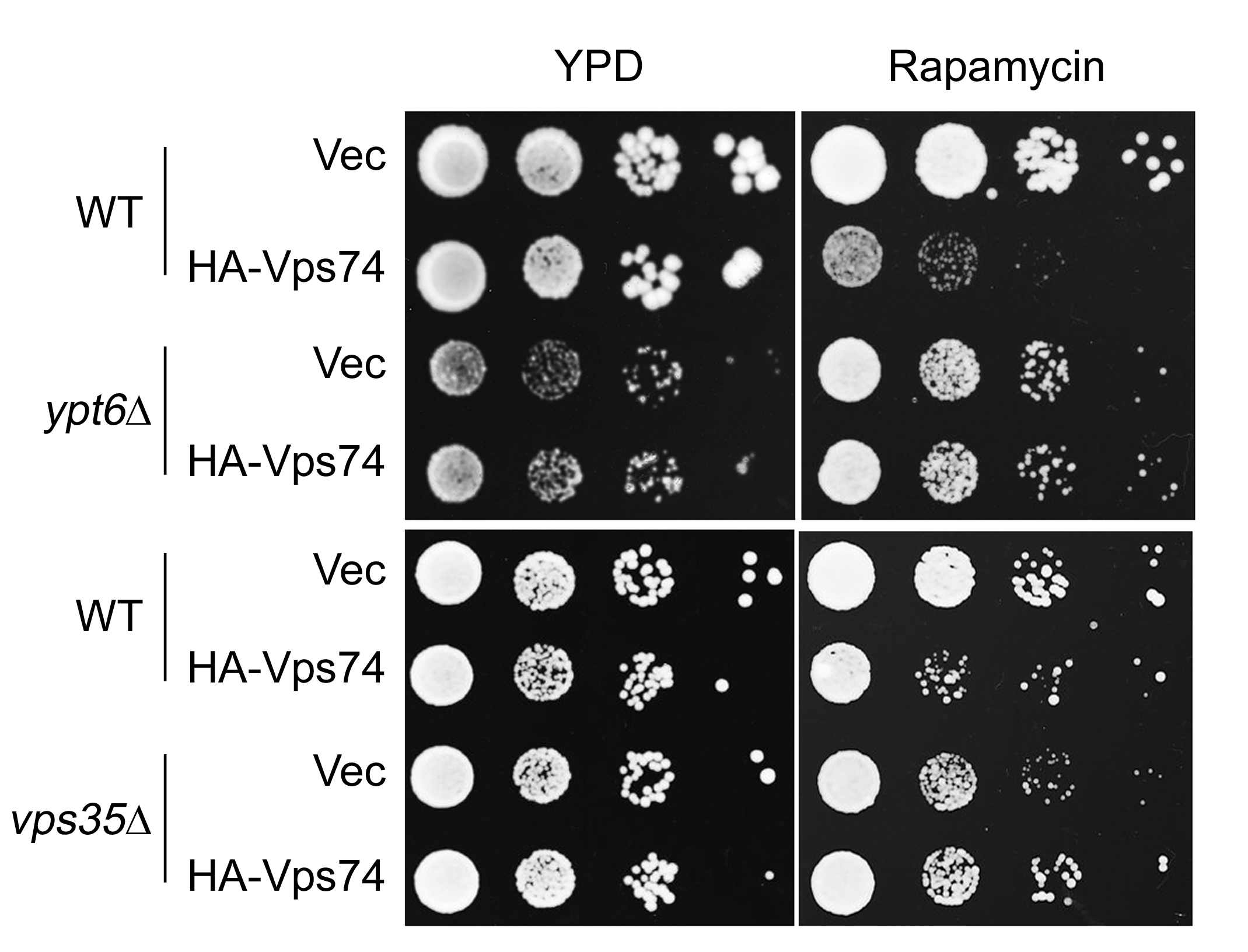

Supplement: Figure S4 — Deletion of YPT6 and VPS35 suppress rapamycin hypersensitivity upon overexpression of Vps74p. Empty vector or Vps74p under an ADH promoter were transformed into wild type, ypt6Δ, or vps35Δ mutant cells. The transformants were serially diluted and spotted on plates of YPD with (right panel) and without (left panel) 100 nM rapamycin to examine their hypersensitivity. (TIF) [file pone.0074715.s004.tif]
