# Supplementary material for: The N-Terminus of Vps74p Is Essential for the Retention of Glycosyltransferases in the Golgi but Not for the Modulation of Apical Polarized Growth in Saccharomyces cerevisiae
Source: PLoS One. 2013 Sep 3;8(9):e74715. doi: 10.1371/journal.pone.0074715 (PMC3760917; doi:10.1371/journal.pone.0074715)
Supplement: Table S1 — Yeast strains used in this study. (DOC) [file pone.0074715.s005.doc]

**Table S1. Yeast strains used in this study**

| Strain | Genotype |
| --- | --- |
| BY4741 | *MATa* *his3, leu2, met15, ura3* |
| BY4741*vps74*Δ | *MATa* *his3, leu2, met15, ura3, vps74* |
| BY4741*vps35*Δ | *MATa* *his3, leu2, met15, ura3, vps35* |
| BY4741*ypt6*Δ | *MATa* *his3, leu2, met15, ura3, ypt6* |
| BY4741*kre2*Δ | *MATa* *his3, leu2, met15, ura3, kre2* |
| BY4741*gas1*Δ | *MATa* *his3, leu2, met15, ura3, gas1* |
| BY4741*arl1*Δ | *MATa* *his3, leu2, met15, ura3, arl1* |
| BY4741*arf1*Δ | *MATa* *his3, leu2, met15, ura3, arf1* |
| BY4741*arl1vps74*Δ | *MATa* *his3, leu2, met15, ura3, arl1, vps74* |
| BY4741*arf1vps74*Δ | *MATa* *his3, leu2, met15, ura3, arf1, vps74* |
| BY4741*vps74*Δ*/*  *KRE2-GFP* | *MATa* *his3, leu2, met15, ura3, vps74, KRE2-GFP* |
| Jy244 | *MATa* *cdc34-2, ade2, ura2, leu2, his3, trp1, lys2, bar1* |
| Jy244*vps74*Δ | *MATa* *cdc34-2, ade2, ura2, leu2, his3, trp1, lys2, bar1, vps74* |
| Jy244*arf1*Δ | *MATa* *cdc34-2, ade2, ura2, leu2, his3, trp1, lys2, bar1, arf1* |
| Jy244*arl1*Δ | *MATa* *cdc34-2, ade2, ura2, leu2, his3, trp1, lys2, bar1, arl1* |
| Jy244*pep8*Δ | *MATa* *cdc34-2, ade2, ura2, leu2, his3, trp1, lys2, bar1, pep8* |
| Jy244*arl3*Δ | *MATa* *cdc34-2, ade2, ura2, leu2, his3, trp1, lys2, bar1, arl3* |
| Jy244*ypt6*Δ | *MATa* *cdc34-2, ade2, ura2, leu2, his3, trp1, lys2, bar1, ypt6* |
| Jy244*sac1*Δ | *MATa* *cdc34-2, ade2, ura2, leu2, his3, trp1, lys2, bar1, sac1* |
| Jy244*gas1*Δ | *MATa* *cdc34-2, ade2, ura2, leu2, his3, trp1, lys2, bar1, gas1* |
| Jy244*mnn*1Δ | *MATa* *cdc34-2, ade2, ura2, leu2, his3, trp1, lys2, bar1, mnn1* |
| Jy244*kre2*Δ | *MATa* *cdc34-2, ade2, ura2, leu2, his3, trp1, lys2, bar1, kre2* |
| Jy81 | *MATa leu2, his3, bar1, pre1-1, pre4-1* |
| Jy81*vps74*Δ | *MATa leu2, his3, bar1, pre1-1, pre4-1, vps74* |
| Jy81*sac1*Δ | *MATa leu2, his3, bar1, pre1-1, pre4-1, sac1* |
| JJY240 | *MATa* W303a background, *sst1, cdc53-1* |
| JJY240*vps74*Δ | *MATa* W303a background, *sst1, cdc53-1, vps74* |
| JJY240*sac1*Δ | *MATa* W303a background, *sst1, cdc53-1, sac1* |
| *cdc4-* | *MATa ade2, ura3, leu2, his3, trp1, cdc4-1* |
| *cdc4-/vps74*Δ | *MATa ade2, ura3, leu2, his3, trp1, cdc4-1, vps74* |
| *cdc4-/sac1*Δ | *MATa ade2, ura3, leu2, his3, trp1, cdc4-1, sac1* |
| *cdc4-/arf1*Δ | *MATa ade2, ura3, leu2, his3, trp1, cdc4-1, arf1* |
| *cdc4-/arl1*Δ | *MATa ade2, ura3, leu2, his3, trp1, cdc4-1, arl1* |
| *cdc4-/gas1*Δ | *MATa ade2, ura3, leu2, his3, trp1, cdc4-1, gas1* |
| *cdc4-/mnn*1Δ | *MATa ade2, ura3, leu2, his3, trp1, cdc4-1, mnn1* |
| *cdc4-/kre2*Δ | *MATa ade2, ura3, leu2, his3, trp1, cdc4-1, kre2* |
| *JY25* | *MATa clb1*::*URA3*, *clb2^ts^*, *clb3*::*TRP1*, *clb4*::*HIS3* |
| *JY25/vps74*Δ | *MATa clb1*::*URA3*, *clb2^ts^*, *clb3*::*TRP1*, *clb4*::*HIS3, vps74* |
| *JY25/sac1*Δ | *MATa clb1*::*URA3*, *clb2^ts^*, *clb3*::*TRP1*, *clb4*::*HIS3, sac1* |
| *JY25/gas1*Δ | *MATa clb1*::*URA3*, *clb2^ts^*, *clb3*::*TRP1*, *clb4*::*HIS3, gas1* |
| *JY25/mnn*1Δ | *MATa clb1*::*URA3*, *clb2^ts^*, *clb3*::*TRP1*, *clb4*::*HIS3, mnn1* |
| *JY25/kre2*Δ | *MATa clb1*::*URA3*, *clb2^ts^*, *clb3*::*TRP1*, *clb4*::*HIS3, kre2* |
| *JY25/arf1*Δ | *MATa clb1*::*URA3*, *clb2^ts^*, *clb3*::*TRP1*, *clb4*::*HIS3, arf1* |
| *JY25/arl1*Δ | *MATa clb1*::*URA3*, *clb2^ts^*, *clb3*::*TRP1*, *clb4*::*HIS3, arl1* |
