# Supplementary material for: The N-Terminus of Vps74p Is Essential for the Retention of Glycosyltransferases in the Golgi but Not for the Modulation of Apical Polarized Growth in Saccharomyces cerevisiae
Source: PLoS One. 2013 Sep 3;8(9):e74715. doi: 10.1371/journal.pone.0074715 (PMC3760917; doi:10.1371/journal.pone.0074715)
Supplement: Table S2 — Plasmids used in this study. (DOC) [file pone.0074715.s006.doc]

**Table S2. Plasmids used in this study**

| Plasmid | Description |
| --- | --- |
| pVT101U | *URA3, 2 µm, ADH1p* |
| pVT101U-HA-VPS74 | *URA3, 2 µm, ADH1p-HA-VPS74* |
| pVT101U-HA-VPS74-dN66 | *URA3, 2 µm, ADH1p-HA-VPS74-dN66* |
| pVT101U-HA-VPS74-dN90 | *URA3, 2 µm, ADH1p-HA-VPS74-dN90* |
| pVT101U-HA-VPS74-dN122 | *URA3, 2 µm, ADH1p-HA-VPS74-dN122* |
| pVT101U-HA-VPS74-dC83 | *URA3, 2 µm, ADH1p-HA-VPS74-dC83* |
| pVT101U-HA-VPS74-3pm | *URA3, 2 µm, ADH1p-HA-VPS74^S14AS19AS23A^* |
| pVT101U-GFP-HA-VPS74 | *URA3, 2 µm, ADH1p-GFP-HA-VPS74* |
| pVT101U-GFP-HA-VPS74-dN66 | *URA3, 2 µm, ADH1p-GFP-HA-VPS74-dN66* |
| pVT101U-GFP-HA-VPS74-dN90 | *URA3, 2 µm, ADH1p-GFP-HA-VPS74-dN90* |
| pVT101U-GFP-HA-VPS74-dN122 | *URA3, 2 µm, ADH1p-GFP-HA-VPS74-dN122* |
| pVT101U-GFP-HA-VPS74-dC83 | *URA3, 2 µm, ADH1p-GFP-HA-VPS74-dC83* |
| pVT101U-GFP-HA-VPS74-3pm | *URA3, 2 µm, ADH1p-GFP-HA-VPS74^S14AS19AS23A^* |
| pVT101U-ARF1 | *URA3, 2 µm, ADH1p-ARF1* |
| pHS12-ARL1-mRFP | *LEU2, CEN, ACT1p-ARL1-mRFP* |
| pHS12-ARF1-mRFP | *LEU2, CEN, ACT1p-ARF1-mRFP* |
